# Supplementary material for: POWR1 is a domestication gene pleiotropically regulating seed quality and yield in soybean
Source: Nat Commun. 2022 Jun 1;13:3051. doi: 10.1038/s41467-022-30314-7 (PMC9160092; doi:10.1038/s41467-022-30314-7)
Supplement: Supplementary file 1 — Supplementary Information [file 41467_2022_30314_MOESM1_ESM.pdf]

POWR1 is a domestication gene pleiotropically regulating seed quality and yield in soybean

Goettel *et al.*

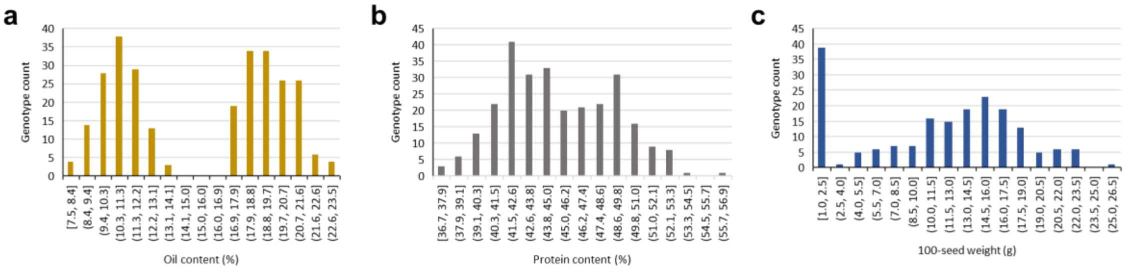

Supplementary Figure 1. Phenotype distribution of the seed traits used for the association studies. **a**, **b**, **and c**, illustrate the phenotypic distribution of seed oil content, protein content, and 100-seed weight, respectively.

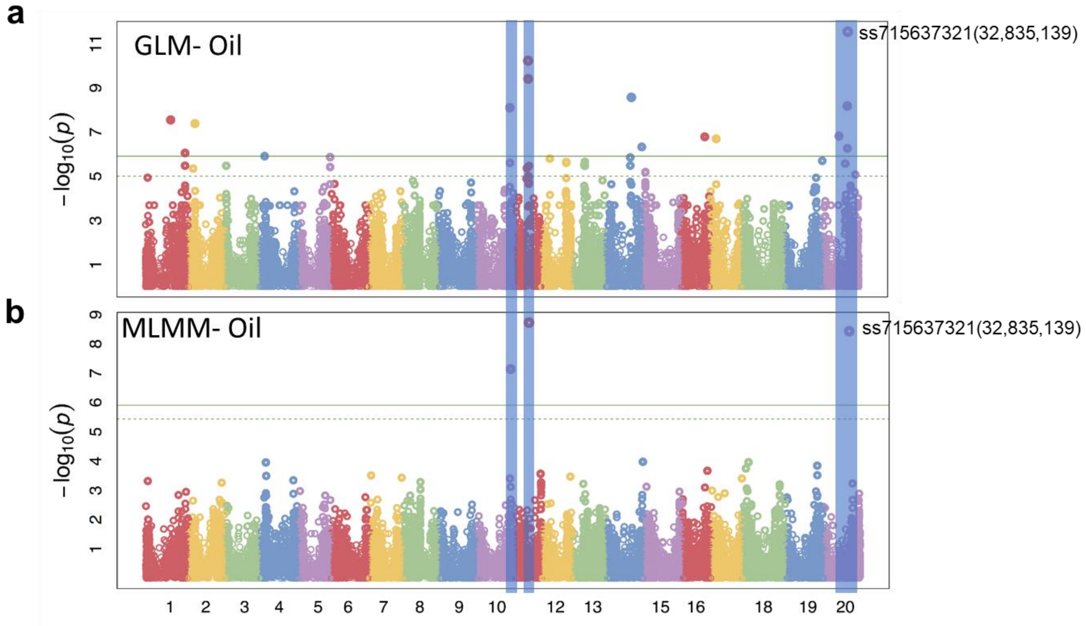

Supplementary Figure 2. GWAS of oil content in the 278 diverse accessions**. a,** GWAS of oil content with GLM model**. b,** GWAS of oil content with MLMM model**.**

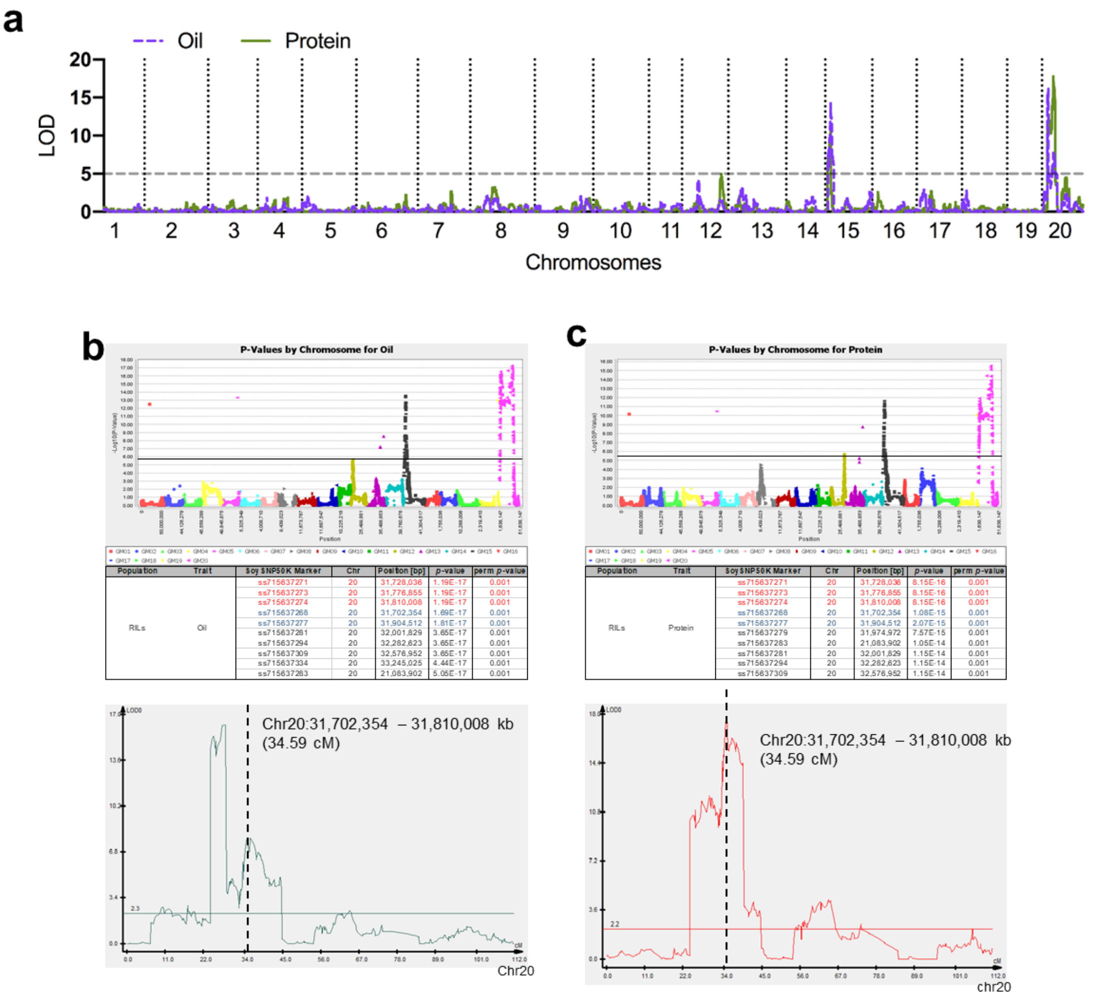

Population	Trait	Soy SNP50K Marker	Chr	Position [bp]	*p*-value	perm *p*-value
RILs	Oil	ss715637271	20	31,728,036	1.19E-17	0.001
		ss715637273	20	31,776,855	1.19E-17	0.001
		ss715637274	20	31,810,008	1.19E-17	0.001
		ss715637268	20	31,702,354	1.69E-17	0.001
		ss715637277	20	31,904,512	1.81E-17	0.001
		ss715637281	20	32,001,829	3.65E-17	0.001
		ss715637294	20	32,282,623	3.65E-17	0.001
		ss715637309	20	32,576,952	3.65E-17	0.001
		ss715637334	20	33,245,025	4.44E-17	0.001
		ss715637283	20	21,083,902	5.05E-17	0.001

Population	Trait	Soy SNP50K Marker	Chr	Position [bp]	*p*-value	perm *p*-value
RILs	Protein	ss715637271	20	31,728,036	8.15E-16	0.001
		ss715637273	20	31,776,855	8.15E-16	0.001
		ss715637274	20	31,810,008	8.15E-16	0.001
		ss715637268	20	31,702,354	1.08E-15	0.001
		ss715637277	20	31,904,512	2.07E-15	0.001
		ss715637279	20	31,974,972	7.57E-15	0.001
		ss715637283	20	21,083,902	1.05E-14	0.001
		ss715637281	20	32,001,829	1.15E-14	0.001
		ss715637294	20	32,282,623	1.15E-14	0.001
		ss715637309	20	32,576,952	1.15E-14	0.001

Supplementary Figure 3. GWAS and linkage mapping of oil content and protein content using 300 RILs. **a** Linkage mapping result of oil and protein content. **b-c,** Association and linkage mapping results of protein and oil content. The most significant associations for both traits are provided below the corresponding Manhattan plot.

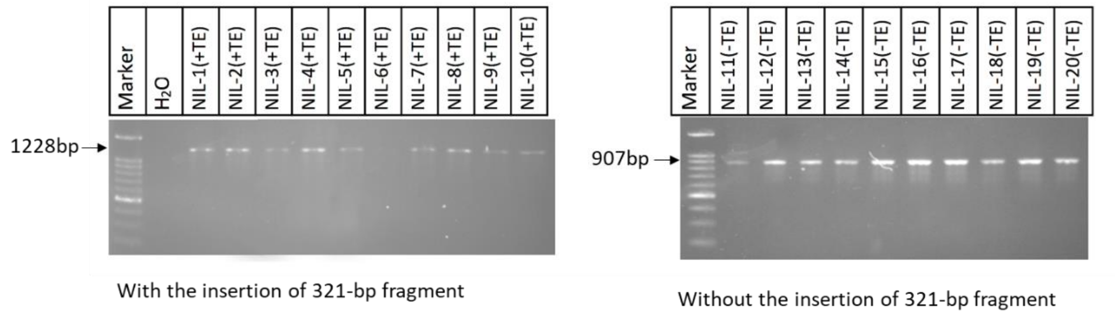

Supplementary Figure 4. PCR-based genotyping of the 321-bp TE in NILs for *POWR1*. NILs show a 1228-bp PCR amplicon with the 321-bp TE insertion while NILs show a 907-bp fragment without the 321-bp TE insertion. This assay was repeated twice.

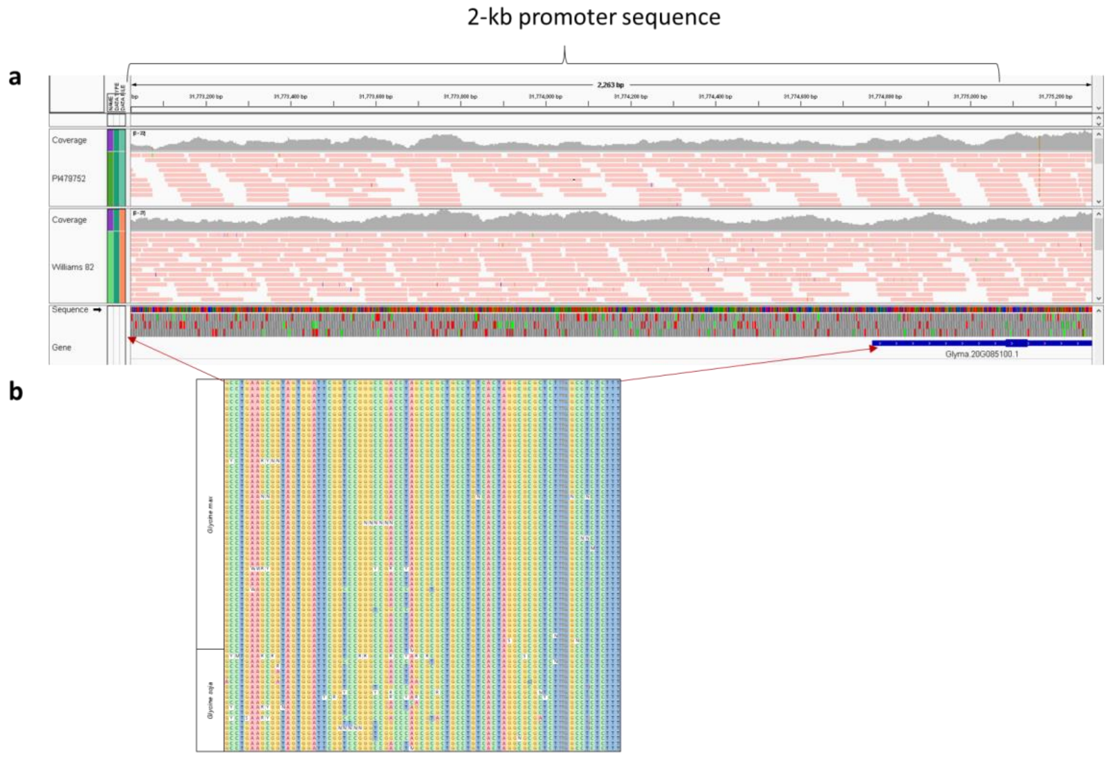

Supplementary Figure 5. Comparison of promoter sequences between two *POWR1* alleles. **a,** IGV visualization of read alignment in the 2-kb region upstream of the start codon of POWR1 in the parental lines of the RIL population, PI479752 and Williams 82. **b,** Sequence comparison reveals nearly identical promoter sequences between two groups carrying *POWR1*$_{\text{-TE}}$ (20 *G. soja* accessions) and *POWR1*$_{\text{+TE}}$ (51 *G. max* accessions). No correlation of seed traits with any DNA variants in their promoters.

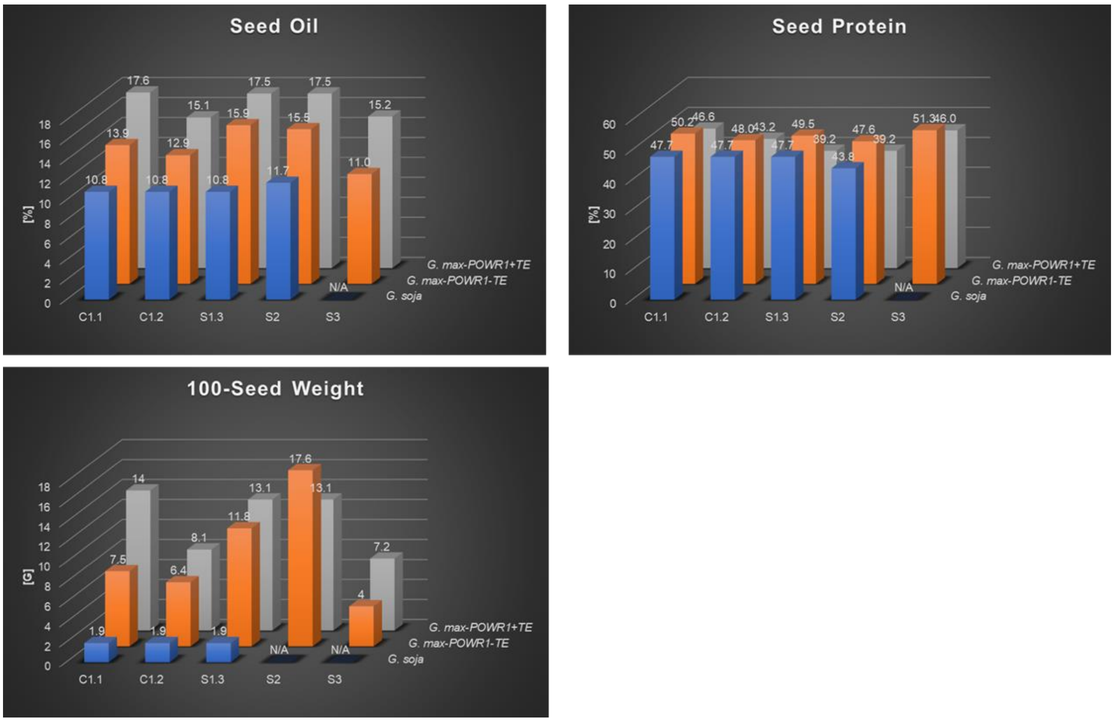

Supplementary Figure 6. Phenotypic changes associated with the transfer of a *POWR1*-TE from *G. soja* into *G. max*. Seed oil content, seed protein content and 100-seed weight of *G. max-POWR1*-TE accessions are compared to their closest *G. soja* accessions and *G. max-POWR1*+TE accessions based on local and global phylogenetic analyses (**see Fig. 4**). The average phenotype values for the Korean clusters 1.1 (C1.1) and 1.2 (C1.2) are given. Both S1.3 and S2 only contain one Japanese accession. A representative accession for S3 is shown. NA: data not available.

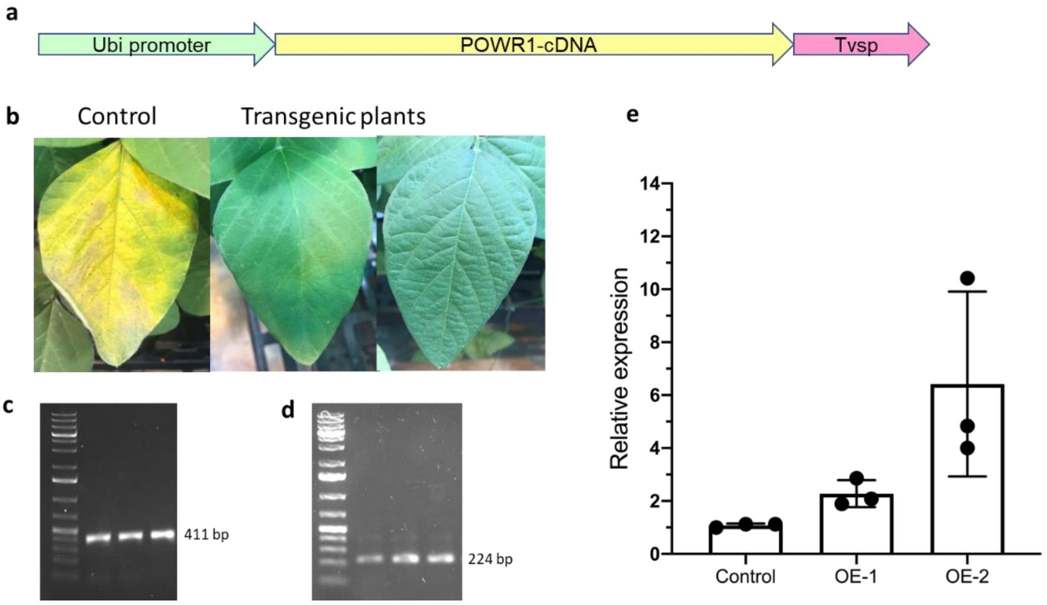

Supplementary Figure 7. Identification of positive transgenic plants by Basta leaf painting assay. **a**, Schematic illustration of the construct (*Ubi917::POWR1*) that was used for overexpression of *POWR1*-TE in soybean. **b**, Basta leaf painting assay showed basta resistance in two transgenic lines and yellowish wilting leaves in control plants. **c** and **d**, PCR verification of three positive transgenic plants using *bar*-specific and *POWR1*-cDNA-specific primers. This assay was repeated twice. **e**, Relative seed expression of *POWR1* in control and two transgenic plants (n=3). Error bars represent SD (standard derivation). Source data are provided as a Source Data file.

a

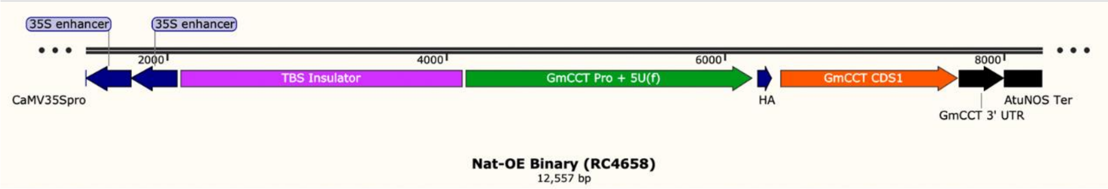

b

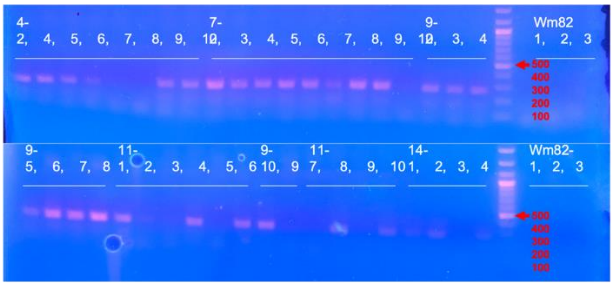

Supplementary Figure 8. Vector diagram and transgenic plant confirmation. **a**, Diagram for the vector used for transformation. **b,** PCR examination for selected lines containing native promoter-driven *POWR1*-TE. PCR produced 266bp in transgenic plants, but not in non-transformed soybean. Wm82 plants is used as a negative control. This assay was repeated twice.

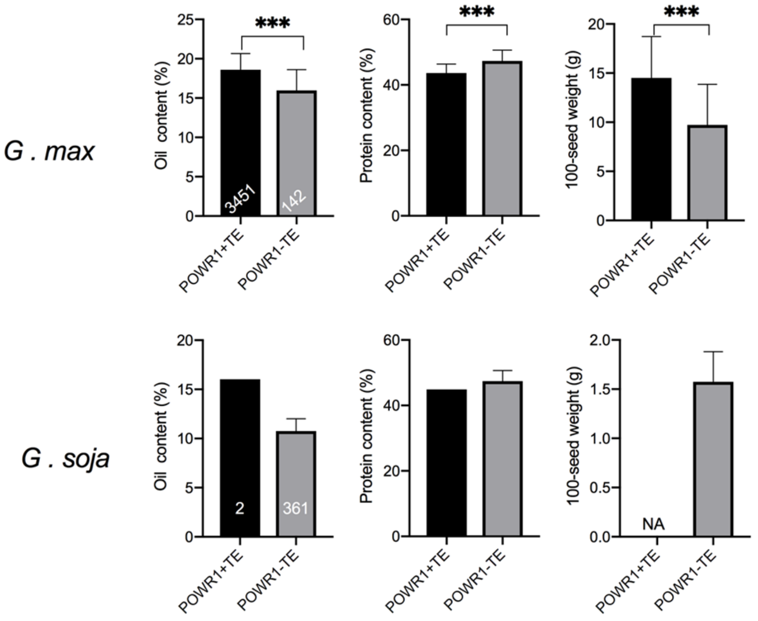

Genotype	Number	Percentange
G. max-*POWR1* +TE	3,451	96.05%
G. max-*POWR1* -TE	142	3.95%
Subtotal	3,593	100.00%
G. soja-*POWR1* +TE	2	0.55%
G. soja -*POWR1* -TE	361	99.45%
Subtotal	363	100.00%
Total	3,956	

Supplementary Figure 9. Frequency of *POWR1* alleles in a diverse population consisting of 3,956 accessions and the allele effects on protein, oil and seed weight from analyzing their whole genome resequencing data.

Supplementary Table 1. Genotyping analysis of the 321-bp InDel in 30 RILs and their parental lines.

Common Name	TE insertion	Oil content [%]	Protein content [%]
PI479752	no	9.6	44.4
I1376	no	11.3	46.0
I628	no	11.5	44.2
I976	no	11.5	48.0
I695	no	11.9	46.5
I1338	no	12.0	46.9
I828	no	12.1	46.8
I668	no	12.2	44.8
I1375	no	12.3	46.7
I680	no	12.3	44.2
I1530	no	12.5	45.6
I144	no	13.2	46.4
I1593	no	13.6	46.2
I665	no	13.6	46.1
I1630	no	13.7	46.4
I200	no	15.3	38.8
I306	yes	15.9	39.0
I646	yes	16.1	38.4
I1652	yes	16.3	38.8
I1342	yes	16.7	38.9
I1490	yes	17.1	38.7
I684	yes	17.5	41.2
I477	yes	17.6	39.9
I32	yes	17.6	40.6
I1234	yes	17.6	39.0
I347	yes	17.7	41.3
I333	yes	17.8	38.6
I1596	yes	18.2	37.9
I259	yes	18.3	39.4
I113	yes	18.3	39.7
I581	yes	18.6	37.6
Williams82	yes	20.5	39.3
Average	no	12.4	45.5
Average	yes	17.6	39.3
Difference between average contents	-	5.2	6.2
p-value (T-test)	-	4.00E-13	3.53E-10

Supplementary Table 2. Primers used in this study.

**Primer name**	**Forward** (5’->3’)	**Reverse** (5’->3’)
Bar gene	AAGCACGGTCAACTTCCGTA	AAGTCCAGCTGCCAGAAACC
POWR1-PCR	TGGCACCAGAAATCTCACACT	TCCTTGTTTATGGCTCTCTCCA
NIL-PCR	CACTTCAAGGGTGGCAGTGTT	CGGGATGGGAAAAGTGTCCTA
AAP1_qRT	TGGTTGCCCTTGGGAATGTGTG	TGGTTGGGCAAGCACCTGATATG
SUC2_qRT	AAAGGCTGCAGAACATGAACGC	GCAGCAGCTTTGACACCAAGTG
SUS4_qRT	AATCTCCCAGGGTGGACTCAAG	GGCGTTCAAGATTGGTCACATGC
GmWRI1a_qRT	ACTTGGTGGGCATGTTTGATAGTG	AGTCTCATCACCAGGTTGAGTGC
GmABI3b_qRT	TGTACTGGCAGGCTATGATTGGC	TGACAATTCTGTGTCTGCATGGC
ABI5_qRT	GGCTCATTATCTGTCCCTCCT	CCCAAGTGTTTGTTGCCTTT
GmOLEO1_qRT	AGGCGCGTGAGATCAAGGACTATG	GCGTGCACACGATTAAGAAGCC
GmDGAT1A_qRT	ACTGAAGGTGCACGTGGATAAGC	GGAAATCCTTCAGAGCAGCCACTG
BCAT-2_qRT	GACTGGTGCCCAAAGAATGTGC	GGAACCCAACGCTTATTAGCCAAG
AAE5_qRT	AGGAGGGTGTGTGATGTTAGGC	CCAACCGTTCTTGAAACAACTTGC
GPAT9_qRT	GATCATGTCCTGGGAGCTAACAAC	TTGTGCAGCCAAGTTCAAACGC

Supplementary Table 3. Information of accessions used for RNA-Seq and *POWR1* expression levels.

PI	Common name	Species subspecies	TE insertion	FPKM	Average FPKM	StDev
PI518671	Williams 82	*Glycine max*	yes	1.010692	0.6679555	0.18984621
PI540556	Jack	*Glycine max*	yes	0.724321		
PI548348	Illini	*Glycine max*	yes	0.506646		
PI548631	Williams	*Glycine max*	yes	0.673904		
PI548656	Lee	*Glycine max*	yes	0.589683		
PI598124	Maverick	*Glycine max*	yes	0.502487		
PI647962	R95-1705	*Glycine max*	no	0.847271	0.9701485	0.28815252
-	JL275	*Glycine max*	no	0.632052		
-	LLL05-14	*Glycine max*	no	1.282608		
-	S09-2902-145	*Glycine max*	no	1.118663		
