# Supplementary material for: POWR1 is a domestication gene pleiotropically regulating seed quality and yield in soybean
Source: Nat Commun. 2022 Jun 1;13:3051. doi: 10.1038/s41467-022-30314-7 (PMC9160092; doi:10.1038/s41467-022-30314-7)
Supplement: Supplementary file 3 — Description of Additional Supplementary Files [file 41467_2022_30314_MOESM3_ESM.pdf]

Description of Additional Supplementary Files

File Name: Supplementary Data 1
Description: Information for all soybean accessions used in this study.

File Name: Supplementary Data 2
Description: SNPs significantly associated with seed protein and oil content and 100-seed weight.

File Name: Supplementary Data 3
Description: TE genotypes of accessions used for biparental QTL mapping experiments.

File Name: Supplementary Data 4
Description: Differentially expressed genes identified by RNA-Seq.
