# Supplementary material for: POWR1 is a domestication gene pleiotropically regulating seed quality and yield in soybean
Source: Nat Commun. 2022 Jun 1;13:3051. doi: 10.1038/s41467-022-30314-7 (PMC9160092; doi:10.1038/s41467-022-30314-7)
Supplement: Supplementary file 8 — Reporting Summary [file 41467_2022_30314_MOESM8_ESM.pdf]

nature research

Corresponding author(s): Yong-qiang Charles An

Last updated by author(s): Mar 21, 2022

Reporting Summary

Nature Research wishes to improve the reproducibility of the work that we publish. This form provides structure for consistency and transparency in reporting. For further information on Nature Research policies, see our Editorial Policies and the Editorial Policy Checklist.

Statistics

For all statistical analyses, confirm that the following items are present in the figure legend, table legend, main text, or Methods section.

n/a	Confirmed	
☐	☒	The exact sample size (*n*) for each experimental group/condition, given as a discrete number and unit of measurement
☐	☒	A statement on whether measurements were taken from distinct samples or whether the same sample was measured repeatedly
☐	☒	The statistical test(s) used AND whether they are one- or two-sided<br>*Only common tests should be described solely by name; describe more complex techniques in the Methods section.*
☒	☐	A description of all covariates tested
☒	☐	A description of any assumptions or corrections, such as tests of normality and adjustment for multiple comparisons
☐	☒	A full description of the statistical parameters including central tendency (e.g. means) or other basic estimates (e.g. regression coefficient) AND variation (e.g. standard deviation) or associated estimates of uncertainty (e.g. confidence intervals)
☐	☒	For null hypothesis testing, the test statistic (e.g. *F*, *t*, *r*) with confidence intervals, effect sizes, degrees of freedom and *P* value noted<br>*Give P values as exact values whenever suitable.*
☒	☐	For Bayesian analysis, information on the choice of priors and Markov chain Monte Carlo settings
☒	☐	For hierarchical and complex designs, identification of the appropriate level for tests and full reporting of outcomes
☒	☐	Estimates of effect sizes (e.g. Cohen's *d*, Pearson's *r*), indicating how they were calculated

Our web collection on statistics for biologists contains articles on many of the points above.

Software and code

Policy information about availability of computer code

Data collection

A panel of 548 soybean accessions (398 cultivated soybean G. max and 150 wild soybean G. soja (Siebold & Zuccarini)) from the genetic resources information network (GRIN) database of U.S. National Plant Germplasm System (https://npgsweb.ars-grin.gov/) was used in this study. Out of 548 accessions, 278 accessions (116 G. soja and 162 G. max) with variations in seed oil (7.5- 23.5%), seed protein (36.7-56.9%) and 100-seed weight (1.0-26.5g) were used for association analysis.

An F6:7 population of 300 recombinant inbred lines (RILs) from a genetic cross between G. max cv. Williams 82 and G. soja PI479752 was used for genetic linkage mapping. Seed oil content among the RILs varied from 9.82–20.47% and 37.64–47.99% for protein content. Seeds of the parents and RILs were planted at the USDA-ARS farms in Beltsville, Maryland, in 2012 and 2015 with two replications in a randomized block design. The highly homozygous (>99%) near-isogenic lines (NILs) were created from a F7 plant heterozygous for POWR1 from a cross of G03-3101 × LD00-2817P.

Plant growth and phenotype measurements were performed as described in 46. The NILs homozygous at the POWR1 locus were planted in replicated field trials in nine environments (one in Arkansas, Missouri, North Carolina, and six in Tennessee) in 2016 and 2017 with randomized complete block design. The TE variations in NIL lines were validated by a PCR assay with a pair of PCR primers flanking the InDel (Supplementary Table 2). All soybean plants including the transgenic lines used for DNA genotyping and quantification of seed traits were grown in the Donald Danforth Plant Science Center greenhouses (St. Louis, MO, USA).

Phenotypic data including seed protein and oil content (%), 100-seed weight (g) for the panel of 548 accessions were acquired from the Germplasm Resources Information Network (GRIN, https://www.ars-grin.gov/). Oil and protein content of the RIL population, the transgenic plants and all other soybean plants were measured using the near-infrared reflectance (NIR) spectroscopy using a DA 7250 NIR analyzer (Perten Instruments, Sweden) unless specified. Approximately 50 seeds per line were analyzed and measured twice. For NILs, approximately 20g seeds were grounded to powder and also measured with Perten DA 7250 analyzer. Seed trait measurements were averaged over all replications and locations for both NIL groups and compared.

Data analysis

All quality-controlled reads were aligned to the G. max reference genome (Williams 82.a2 v1) with BWA (0.7.15). DNA variants including SNPs and InDels were called using the GATKs pipeline. The resulting variants were filtered using GATKs VariantFiltration with following parameters:

read depth ≥ 5 reads, SNP quality ≥ 50, and at least 2 SNPs in a 10-bp window were allowed. Read alignments were visualized using the Integrative Genomics Viewer. The resulting 28,708 SNP and 131 InDel markers in a 4.1-Mb region (29 - 33.15 Mb) were used to carry out regional association analyses. Whole developing seeds at the mid-maturation stage were collected in environmental controlled greenhouses and multiple seeds per accession were pooled for transcriptome sequencing. Transcriptome analysis was performed with TopHat (2.0) and Cufflinks (2.2.1), and the FPKMs across samples were normalized with the quantile method in Cuffdiff.

DNA variants were quality controlled before being used for genome-wide or regional association analysis with TASSEL5 with following criteria: a minimum minor SNP allele frequency of 0.05, a maximum proportion of heterozygous sites of 0.2, and a minimum number of accessions per site of 85%. Five principal components as determined in TASSEL5 were used for population structure (Q). Kinship (K) was calculated using centered IBS method in TASSEL5. GLM (general linear model) and MLMM (mixed linear model) were used for genome-wide association mapping and regional association analysis, as implemented in TASSEL. For the RIL population, GLM without population structure Q, or GLM with Q, or MLM with Q and kinship K returned almost identical mapping associations for oil and protein using 19,848 SNPs from the SoySNP50K-set. The Bonferroni-corrected genome-wide significance threshold was calculated as 0.05/SNP count. Linkage mapping was carried out using Windows QTL Cartographer v2.5 and QTLs were detected using the composite interval mapping with 1,000 permutations for each test.

Principal Component Analysis (PCA) of the association panel was conducted in TASSEL (v5) using the SoySNP50K SNPs. The wild soybean and cultivated soybean accessions from the 548 accessions were used to calculate Tajima's D and the pairwise nucleotide diversity πwas calculated in TASSEL5. Regions accounting for the top 15% ln-ratios (which corresponds to an ln-ratio threshold of about 2.4) or Tajima's D of < -2 were considered as domesticated.

The unrooted Neighbor-Joining phylogenetic tree was constructed with the 548 accessions using MEGA7 with the Maximum Likelihood method based on the Tamura-Nei model. A total of 19,284 genome-wide SNPs were used for the global tree and 1,023 SNPs within the 154-kb domestication region were used for the local tree. Multiple DNA and protein alignments were performed in Clustal Omega (https://www.ebi.ac.uk/Tools/msa/clustalo/). Structures of the proteins were predicted by I-TASSER, were compared with RaptorX (TMscore 0.797) and visualized with iCn3D.

For manuscripts utilizing custom algorithms or software that are central to the research but not yet described in published literature, software must be made available to editors and reviewers. We strongly encourage code deposition in a community repository (e.g. GitHub). See the Nature Research guidelines for submitting code & software for further information.

Data

Policy information about availability of data

All manuscripts must include a data availability statement. This statement should provide the following information, where applicable:

- Accession codes, unique identifiers, or web links for publicly available datasets
- A list of figures that have associated raw data
- A description of any restrictions on data availability

The genomic data supporting the findings of this study are available in the SoyBase (https://soybase.org) and Ag Data Commons (https://doi.org/10.15482/USDA.ADC/1519167). The Williams 82 soybean reference genome sequence was downloaded from the Phytozome v12 (https://phytozome.jgi.doe.gov/pz/portal.html). The SoySNP50K iSelect Bead Chip for reported soybean accessions in the US Soybean Collection were downloaded from the SoyBase. The phenotypic data for reported accessions ara available in Genetic Resources Information Network (GRIN, http://www.ars-grin.gov). The data supporting the results of this study are available from the corresponding authors upon reasonable request. Source data are provided with this paper.

Field-specific reporting

Please select the one below that is the best fit for your research. If you are not sure, read the appropriate sections before making your selection.

☒ Life sciences ☐ Behavioural & social sciences ☐ Ecological, evolutionary & environmental sciences

For a reference copy of the document with all sections, see nature.com/documents/nr-reporting-summary-flat.pdf

Life sciences study design

All studies must disclose on these points even when the disclosure is negative.

Sample size	No statistical methods were used to predetermine sample size. There are at least three biological samples that have been used in similar studies.
Data exclusions	No data was excluded from the analysis
Replication	All experiments were independently performed twice and result were successfully repeated.
Randomization	The plants grown in the greenhouse were randomly organized. The cells in the microscopic assay were randomly selected.
Blinding	The researchers performing RNA-seq were blinded to plant selection.

Reporting for specific materials, systems and methods

We require information from authors about some types of materials, experimental systems and methods used in many studies. Here, indicate whether each material, system or method listed is relevant to your study. If you are not sure if a list item applies to your research, read the appropriate section before selecting a response.

Materials & experimental systems

n/a	Involved in the study
☒	☐ Antibodies
☒	☐ Eukaryotic cell lines
☒	☐ Palaeontology and archaeology
☒	☐ Animals and other organisms
☒	☐ Human research participants
☒	☐ Clinical data
☒	☐ Dual use research of concern

Methods

n/a	Involved in the study
☒	☐ ChIP-seq
☒	☐ Flow cytometry
☒	☐ MRI-based neuroimaging
